# Humerus trochlear angle (HTa)—a possible alternative for Baumann angle in the reduction of supracondylar humerus fractures

**DOI:** 10.1186/s12891-021-04717-4

**Published:** 2021-11-15

**Authors:** Gang Chen, Lu Cui, Jiaqi Shi, Peng Zhang, Jun Li, Zijian Wang, Jun Song, Bangjun Wang

**Affiliations:** Department of Orthopedic Trauma, Xiangyang Central Hospital, Affiliated Hospital of Hubei University of Arts and Science, Jinzhou Road 136, Xiangyang, Hubei People’s Republic of China

## Abstract

**Background:**

The supracondylar humerus fractures are the most common fracture in children’s elbows. Generally, close reduction and percutaneous pinning can provide satisfactory outcomes after adequate reduction. Baumann angle is commonly used to evaluate reduction quality, however, it may fail to assess reduction well when the elbow is in flexion and/or when the patient is young. We conducted this study to evaluate the potential value of the humerus trochlear angle (HTa) for the reduction evaluation and compare it with the Baumann angle.

**Methods:**

We retrospectively reviewed supracondylar humerus fractures in our trauma center from 2016 to 2019. Patients were grouped as followed: in the HTa group, an arthrogram was used to evaluate the HTa angle and reduction (HTa, defined by the intersection of the axis of the humerus shaft and the tangent of the articular surface of the trochlear); In the Baumann group, the Baumann angle was used to assess the reduction. Baumann angle ratio (BA of injured side/BA of contralateral side) was calculated to evaluate the reduction quality between groups. Flynn’s grading criteria were utilized to evaluate both function and cosmetic outcomes in two groups during the follow-up. Operation time, fluoroscopy shots, complications and Flynn’s grading scores were compared between groups.

**Results:**

A total of 57 patients with an average age of 4.62 years and follow-up duration of 21.49 ± 5.40 months were included in the analysis. The gender and age distributions were similar in the two groups. Fluoroscopy shots in the HTa group were significantly less than in Baumann group (16.17 ± 0.73 vs. 21.85 ± 0.78, *p* < 0.0001), and operation time were also less in HTa group (45.78 ± 1.96 min vs. 62.21 ± 1.58 min, *p* < 0.0001). Baumann ratio showed no significant difference between the two groups (1.002 ± 0.023 in the Baumann group and 1.01 ± 0.023 in HTa group, *p* < 0.0001). Length of hospitalization, complications, and Flynn’s grading scores were similar between groups. The HTa angle was positively correlated with Baumann angle in the HTa group (*R*-value is 0.71 and *P* = 0.0002).

**Conclusions:**

There was no significant difference in reduction quality and Flynn’s scores between HTa and Baumann groups. Furthermore, HTa was associated with shorter operation time and less radiation exposure in this investigation. Therefore, HTa may be a convenient and reliable parameter that could guide the reduction of supracondylar humerus fractures, especially for young children.

## Background

The supracondylar humerus fractures are the most common in children’s elbows, and the inadequate reduction usually induced significant complications, such as cubitus valgus and ulnar nerve palsy [1–3]. Currently, recommend treatment strategies, including close reduction and percutaneous pinning, can decrease the risk of malunion and compartment syndromes [2, 4–6]. However, for a junior pediatric orthopedic surgeon, it remains a challenge in the close reduction, especially when the patient is a young child with small ossification nuclei [7, 8]. Since the un-matured epiphysis is radiolucent, it is difficult to identify the contour of the distal humerus and to assess the reduction, especially when the elbow is in flexion and the interference of the forearm makes the measurement of Baumann angle more difficult [9, 10]. Therefore, it is critical to obtain an alternative parameter for assessing the reduction in Jones’ view, particularly for young children.

An arthrogram is initially developed as a novel technique to assess the cartilage injury of joints when CT or MRI is not available, which is designed to make the articular surface visible under fluoroscopy by the injection of an appropriate contrast agent into the joint [11]. With the advantage of minimal invasion, arthrogram has been successfully employed in series of investigations, such as radial head fractures and lateral condylar fractures in children [12–14].

**Fig. 1:**
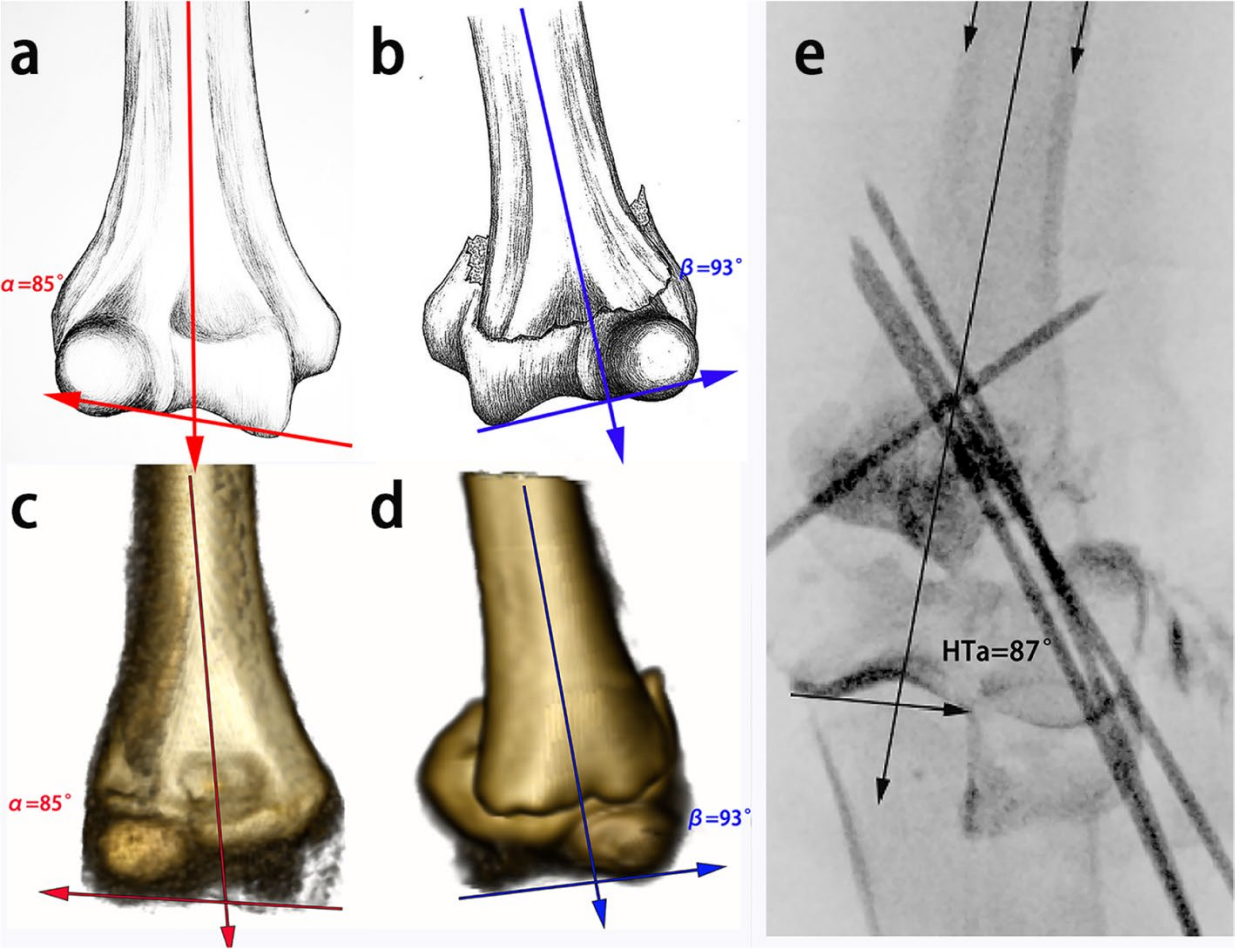
The humerus trochlea angle (HTa) was defined by the intersection of the axis of humeral shaft and the tangent to the curve surface of trochlea: for example, on the contralateral side of a patient in HTa group, the HTa (red) was 85° (**a**, **c**); on the injured side, the HTa (blue) was 93° (**b**, **d**); after reduction with arthrogram, the HTa (black) was restored to 87° (**e**). Three-dimensional models in (**c**) and (**d**) were provided from DICOM by an open source medical image viewer (Horos V 3.3.5., Swiss); all angle measures were performed by RulerSwift (Mac OS X free soft)

Generally, in our department, if the patient is suspicious of a supracondylar humerus fracture combined with possible epiphysis injury, an arthrogram would be performed. With help of an arthrogram, it is convenient to identify the contour of the distal fracture segment and to evaluate the fracture reduction. Furthermore, in patients with Garland type I fracture and patients with type II/III after perfect reduction, we noticed that the axis of the humerus may intersect with the lateral third of the articular surface of the trochlea, forming an angle (humerus trochlea angle, HTa) (Fig. 1). Thus, we could utilize HTa to evaluate the reduction quality. This angle was defined by two lines (i.e. the humerus axis and the tangent at the prominent surface of the trochlea) is generally less than 90°, which may expand the angle when fractures are not corrected completely (Fig. 1). Due to the advantage of arthrogram, HTa was more clear and convenient than Baumann angle when elbow in flexion. In previous studies, the measure of Baumann angle might be interfered with by the forearm if elbow in flexion [15, 16].

Thus, we supposed that HTa may be a possible alternative parameter for Baumann angle, which may guide the reduction of supracondylar humerus fractures, especially when patients are young or the ossification nuclei is too obscure to be identified. In addition, we supposed that the HTa angle may be correlated with the Baumann angle to some degree. The main aim of the present study is to evaluate the potential value of HTa in the reduction of supracondylar humerus fractures in young children.

## Methods

A retrospective review was performed with the approval of the hospital institutional review board of the Xiangyang Central Hospital. All procedures were performed in accordance with the Declaration of Helsinki. Patients’ medical records were searched using the hospital information system (HIS, Neusoft Corporation, China) from January 2016 to December 2019 in our orthopedic trauma center.

Include criteria of this investigation were as follows: (1) age ≤ 7 years old; (2) Garland type III supracondylar humerus fracture; (3) close fracture; (4) treating with close reduction and percutaneous pinning in supination. Additionally, exclude criteria included: (1) Garland type I/II supracondylar humerus fractures; (2) open fractures; (3) combined with other fractures or nerve and vascular injuries; (4) treating with open reduction and fixation; (5) loss occurred during follow-up.

All patients underwent X-ray exams in anteroposterior (AP) view and lateral view before, in, and after the operation. All radiographic results were collected in Picture Archiving and Communication Systems (PACS, Neusoft Corporation Ltd, China) and assessed by two independent senior orthopedic surgeons twice. The data means were calculated for further analysis.

In this investigation, some key parameters were defined as follows:

1. Baumann angle is defined by the intersection of the longitude axis of the humerus shaft and the line between the humerus capitellar ossification nuclei and humerus metaphysis, which can be measured when the radiation beam is perpendicular to the anterior part of the elbow, with the full elbow extension and without any forearm rotation [15–17].
2. Furthermore, the Baumann angle ratio (i.e. Baumann angle of the injured side / Baumann angle of the contralateral side) is calculated to evaluate the reduction quality on the coronal plane. Due to the Baumann angle from each side of the same patient is similar, theoretically, this ratio may approach 1.0 if the fracture sustained a perfect reduction.
3. HTa is defined by the intersection of the humerus shaft axis and the tangent to the curved surface of the trochlea (Fig. 1).

According to the involvement of arthrogram, patients were divided into two groups, including the Baumann group of 34 cases without arthrogram and the HTa group of 23 cases with an arthrogram. There were some differences in the reduction and pinning strategies between the two groups.

In the Baumann group, patients were laid in supination with an injured elbow right over the fluoroscopy beam. After the slow and consistent traction, the fracture would be reduced. Subsequently, the elbow needed to be flexed and the Baumann angle could be measured in Jones’ view for assessing the alignment on the coronal plane. Furthermore, the alignment on the sagittal plane could be measured in the lateral view based on the location of the anterior humerus line (AHL) [18].

The first pin could be inserted only when the reduction fulfilled the standard of the American Academy of Orthopedic Surgeons (AAOS) [2, 19]. Then, the surgeons would extend the elbow and check the alignment on the coronal plane again.

In some cases, Baumann angle detected in AP view with the elbow fully extended may be more accurate than that in Jones’ view due to the measurement is not be affected by the forearm (mainly the proximal parts of ulnar and radius when elbow in flexion) [15, 16]. Furthermore, if the Baumann angle is adequate (identical to the contralateral side), the following pins can be inserted in sequence [5, 20, 21]. If not, the initial pin should be removed and the reduction needs to be performed again.

In the HTa group, the arthrogram was performed as previously described (0.5 ml -1.0 ml, Iodixanol, GE Healthcare, Ireland) [11–13]. The process of reduction and pinning was consistent with the Baumann group, except for the utilization of HTa in the assessment of reduction.

HTa can be directly measured in AP view even if the elbow flexes to 130 degrees (Jones’ view), which is a more stable position for the reduction before pinning in most extension type fractures. Then, the first pin can be inserted after HTa is assessed and valid in Jones’ view and sagittal alignment is valid in lateral view. The remaining pines were inserted after verifying HTa angle and AHL with elbow flexion.

To decrease the inter-observer and intra-observer errors, the lines and angles were drawn and measured by two senior surgeons twice, and the means were obtained as the final result.

### Follow-up

The injured elbow needed to be immobilized in plaster for 3 weeks, and the pins would be removed within 3–6 weeks after radiogram estimation. During follow-up, for each patient, an X-ray examination was conducted in the first 3 months and last reviews and physical examination was performed at each time, including function outcomes evaluation and cosmetic outcomes assessing. Additionally, any complications would be recorded.

### Statistical analysis

Results were presented as means ± standard deviation. Graphpad Prism 8.0 (GraphPad Software ltd, San Diego, CA, USA) was utilized for all statistical analyses. *P* < 0.05 was defined as statistically significant. Fisher’s exact test was used to analyze the distribution difference of age and gender and ANOVA was used to analyze the difference of Baumann angle between the two groups. The linear regression was utilized to analyze the potential association between HTa and Baumann angle in the HTa group.

## Results

Total 57 cases were included in the final analysis, with 34 cases in the Baumann group and 23 in the HTa group. The gender and age distributions in each group were listed in Table 1. There were 20 boys and 14 girls in the Baumann group, with 11 boys and 12 girls in the HTa group. The mean age in the Baumann group was 4.8 years old (range from 1.2 to 7 years), with 4.3 years old in the HTa group (range from 2 to 6.2 years), and there was no significant difference in age between the two groups (*p* = 0.1458). Furthermore, there was no significant difference in inpatient time between the two groups (4.62 ± 0.25 days vs. 4.70 ± 0.27 days, *p* = 0.83).

**Table 1:** General patients data

	Baumann group	HTa group	*P* value
Sex	34	23	
Male	20	11	*P* = 0.4323
Female	14	12	
Age (year)	4.83 ± 0.22	4.31 ± 0.28	*p* = 0.1458
Operation time (min)	62.21 ± 1.58	45.78 ± 1.96	*p* < 0.0001
X-ray exposure (shots)	21.85 ± 0.78	16.17 ± 0.73	*p* < 0.0001
Follow-up time (months)	20.41 ± 1.04	22.22 ± 1.23	*p* = 0.2699
Inpatient time (days)	4.62 ± 0.25	4.70 ± 0.27	*p* = 0.835

The Baumann angle ratio was calculated to assess the reduction quality on the coronal plane between the two groups. In the Baumann group, the ratio mean was 1.002 ± 0.023, and that was 1.01 ± 0.023 in the HTa group. Therefore, there was no significant difference in the Baumann angle ratio between the two groups (*p* < 0.0001), which indicated that although different procedures were used, these fractures still acquired similar reduction quality on the coronal plane.

The mean duration of follow-up was 20.41 ± 1.04 months in the Baumann group, and 22.22 ± 1.23 months in the HTa group. The elbow function (loss of movement range) and the cosmetic scores (loss of carrying angle) were measured by a protractor during follow-up, and these results were calculated and analyzed according to Flynn’s grade system [5] (Table 2). Total 29 cases (85.29%) in the Baumann group showed excellent results, 3 (8.82%) cases showed good results, and 2 (5.88%) showed fair results. Additionally, the results in the HTa group were as follows: 20 (86.96%) in excellent, 2 (8.70%) in good, and 1 (4.35%) in fair. No case sustained poor results in both groups.

**Table 2:** Comparing of radiography results, function outcomes and complications

	Baumann group		HTa group	
Baumann angle				
Injury side	68.5 ± 3.16		70.04 ± 3.82	
Contralateral side	68.68 ± 3.36		70.74 ± 3.67	
Complications				
Infection	3		2	
Ulnar nerve palsy	3		1	
Reduction loss	1		2	
Results degree	Number of case	Ratio(%)	Number of case	Ratio(%)
Excellent	29	85.29	20	86.96
Good	3	8.82	2	8.70
Fair	2	5.88	1	4.35

Besides, we also investigated the association between HTa and Baumann angle in the HTa group. The results indicated that HTa was positively associated with Baumann angle on the injured sides (linear regression in PRISM was utilized for the analysis, and F = 0.67, Y = 0.3874*X + 37.4).

## Discussion

The supracondylar humerus fracture is the most common elbow fracture in children [5, 22, 23]. Inadequate reduction frequently induced malunion, especially cubitus valgus, a lifetime deformity that was hazardous for children’s physical and mental health [23], and other complications may also occur, e.g. ulnar nerve injury, compartment syndrome, and elbow stiffness [24, 25].

In most investigations, close reduction and percutaneous pinning as the first choice were considered for Garland type II and type III fractures to obtain excellent/good results [5, 14, 21, 24, 25]. Nonetheless, for a junior orthopedic surgeon, it remains a challenge due to patient is young and un-matured distal humerus epiphysis is obscure to be identified, although guiding parameters (e.g. Baumann angle) may provide some help [19].

Despite the Baumann angle, other various parameters were also investigated in previous studies, such as humerus-ulnar angle and metaphyseal-diaphysis angle [8, 26]. Among these parameters, the Baumann angle is the most reliable and accurate on the coronal plane [16, 27, 28]. On the sagittal plane, the anterior humerus line (AHL) was the most reliable guiding parameter in reduction [18].

However, there are still underlying factors that may affect the accurate measurement of Baumann angle in clinical practice, such as, the tilting and the rotation of distal fragment may induce the change of Baumann angle [15, 16]. Besides, the projection angle of the fluoroscopy beam can also cause the measurement deviation of Baumann angle [17], and the physiological changes and deviations of epiphysis ossification nuclei are obstacles to accurate measurement [8].

For example, the capitellar ossification nuclei usually appear at age of 2 years, and at 5 to 7 years old and 8 to 9 years old, the medial epicondyle ossification nuclei and the trochlear ossification will present, respectively [29], which make it more difficult to identify distal humerus with fluoroscopy. In young children (≤ 7 years old), because the distal humerus ossification nuclei are small and round, limited information about the contour of the articular surface of the elbow joint can be obtained under fluoroscopy. Therefore, the measure of the Baumann angle may interfere in these cases. Hence, investigations of further reliable and accurate parameters are critical clinically.

Additionally, the flexion of the elbow made the measure of the Baumann angle more difficult. In a standard close reduction procedure, it was recommended to assess the alignment on the coronal plane in Jones’ view before pinning, due to the reduction in this position was more stable [22, 30, 31]. But, the measure of Baumann angle was usually more convenient and accurate in a “true” AP view (with the elbow in full extension and the forearm in full supination), although the fracture in this position is more unstable and easy to displace again [15, 16]. Thus, it is difficult to achieve simultaneously the accurate measurement of Baumann angle and satisfactory reduction, which is a primary dilemma in clinical practice. Sometimes, surgeons were recommended to check the Baumann angle again with the elbow in extension right after the first pinning; and if the reduction and angle were not appropriate, which occasionally occurred, the first pin should be removed and the reduction-checking-pinning cycle needed to be performed again [5, 20, 21]. It was significantly time-consuming and partly hazardous, due to the repeated pinning may hurt the blood supply of epiphysis [20, 32, 33], and the revised reduction and pinning contributed a lot to longer operation time and more radiation exposures to patients.

Recently, we noticed that the HTa angle was significantly steady and less than 90° in the ‘normal’ elbow in young children (‘normal’ indicated no fracture or Garland type I supracondylar humerus fractures or severe fractures but sustained perfect reduction). In the patient with a significantly medial displaced supracondylar fracture, the HTa angle was more than 90 degrees before reduction. Thus, we purposed that HTa angle might be steady and correlate with Baumann angle in the elbow and had the potential of aiding in a close reduction in supracondylar humerus fracture as Baumann angle did.

In the present study, we measured the Baumann angle of both sides in each group. Additionally, we compared the Baumann angle ratio of each group and found no significant difference between the injured side and the contralateral side in both groups. These results indicated that the reduction guided by the HTa angle in the HTa group was as accurate as the reduction of the Baumann group. In addition, the elbow function and the cosmetic scores measured during follow-up showed that no significant difference existed between the HTa group and the Baumann group. Thus, it could be concluded that the reduction quality in these two groups was similar in this investigation.

Besides these radiogram parameters, the operation time, radiation exposure, and inpatient time were investigated and compared. Although the additional arthrogram was performed, the operation time in the HTa group was significantly less than that in the Baumann group (45.78 ± 1.96 min vs. 62.21 ± 1.58 min, *p* < 0.0001). Meanwhile, the total radiation exposure (measured as fluoroscopy shots) showed the same tendency between the two groups (16.17 ± 0.73 shots vs. 21.85 ± 0.78 shots, *p* < 0.0001). The present findings suggested that HTa defined by stable anatomical landmarks is convenient and visible directly with the aid of arthrogram.

**Fig. 2:**
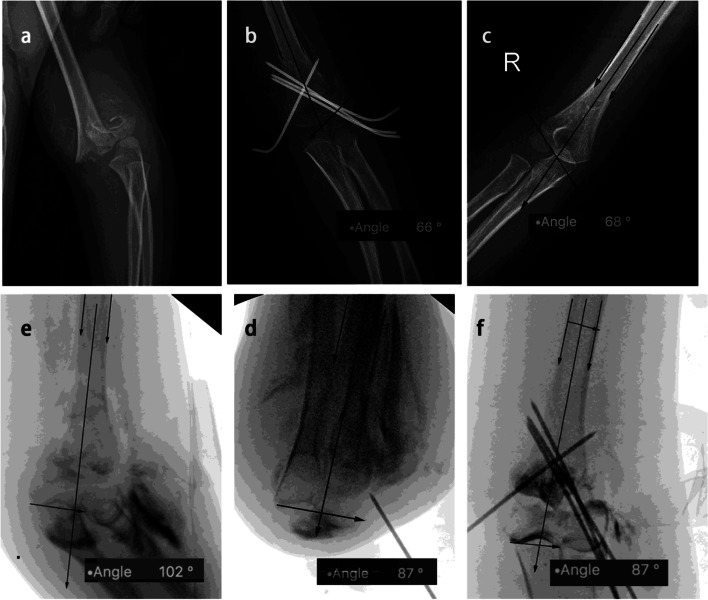
The upper panel showed AP view of a right supracondylar humerus fracture (Garland type III) in HTa group before and after reduction (**a**, **b**), and contralateral side (**c**); the Baumann angle of both sides were 66° vs. 68°. The lower panel illustrated the reduction and pinning process under fluoroscopy and arthrogram, indicating the HTa angle decreased from 102° to 87° (**e**, **d**, **f**) after reduction

**Fig. 3:**
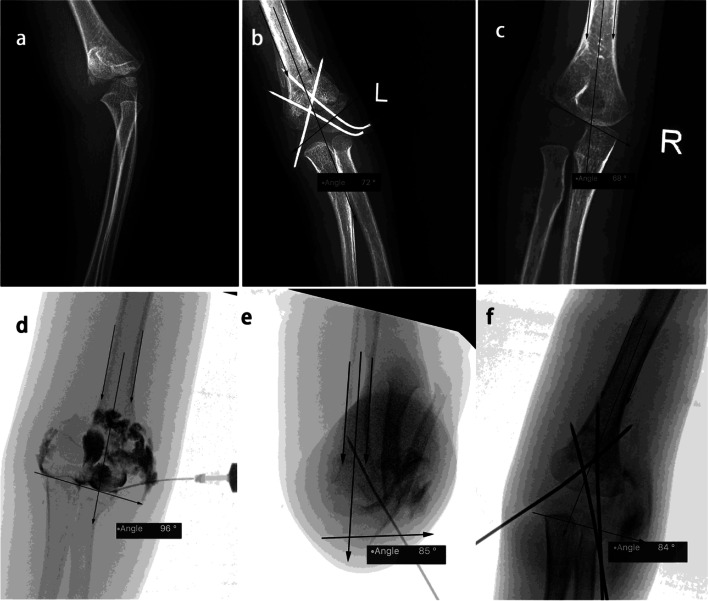
The upper panel showed AP view of a left supracondylar humerus fracture (Garland type III) in HTa group before and after reduction (**a**, **b**), and contralateral side (**c**); the Baumann angle of both sides were 72° vs. 68°. The lower panel illustrated the reduction and pinning process under fluoroscopy and arthrogram, indicating the HTa angle decreased from 96° to 84° (**e**, **d**, **f**) after reduction

In AP view, the articular surface of the trochlea was easy to be identified using the arthrogram; and the tangent at the curve of the articular surface can be drawn conveniently (Fig. 1). Despite that, the angle could be measured easily with the elbow in flexion, while in this position, the measure of Baumann angle would be more difficult (Figs. 2 and 3).

**Fig. 4:**
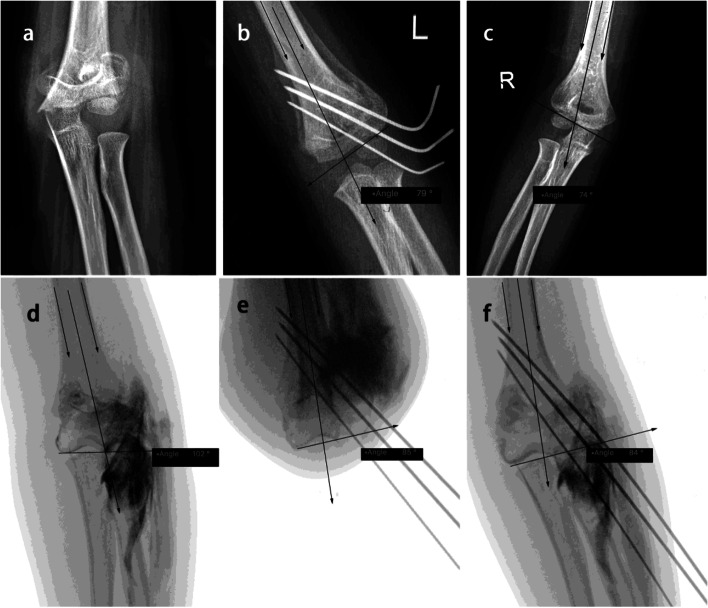
The upper panel showed AP view of a left supracondylar humerus fracture (Garland type III) in HTa group before and after reduction (**a**, **b**), and contralateral side (**c**); the Baumann angle of both sides were 79° vs. 74°. The lower panel illustrated the reduction and pinning process under fluoroscopy and arthrogram, indicating the HTa angle decreased from 102° to 84° (**e**, **d**, **f**) after reduction

The utilization of HTa made a more convenient reduction protocol possible: slow traction with the elbow in extension to verify coronal alignment in AP view, then flex the elbow gently to verify the sagittal alignment in lateral view. Subsequently, the HTa was utilized for a quick and convenient assessment for the coronal alignment, with the elbow in flexion. After verification on two planes, the remained pins were then performed (Fig. 4).

Some investigators preferred to insert the first pin to fix the reduction temporally, and then assess the Baumann angle with the elbow in extension. Next, the remaining pins would be inserted if the alignment was verified. If not, the initial pin must be removed and the reduction needs to be performed again. We presumed this difference in protocol was the main cause of prolonged operation time in the Baumann group.

In addition, the present study detected that the larger the HTa angle, the more the Baumann angle. The present analysis in PRISM provided significant results (*R*-value is 0.71 and *P* = 0.0002) and indicated that the HTa angle of the injured side was positively correlated with the Baumann angle of the same side after reduction. It was contributed to the reasons below: at the first, Baumann angle was generally correlated with the carry angle in children [16]; secondly, the carry angle was complementary with the angle formed by the humerus axis and ulnar axis; in the end, the angle was the angle formed by humerus axis and trochlea surface which was almost perpendicular to the ulnar axis. Taken together, the correlation of HTa angle and the carry angle or Baumann angle was interpretable and acceptable, which was also further verified by the line regression analysis in PRISM.

There were several limitations in the present study. Firstly, the retrospective study provides only some weak evidence, and a randomized multicenter clinical study may be more powerful. Secondly, the sample size of the present study was small. The anatomical characters of the trochlea surface and the normal range of HTa angle will be more accurate if the sample size is large enough. Thirdly, due to the deficiency of equipment, the radiation exposures were calculated only using the means of fluoroscopy shots rather than radiation dose, which may lead to inaccuracy, to some degree. Finally, the intra-observer and inter-observer errors need to be considered in future study.

## Conclusion

There was no significant difference in the reduction quality and Flynn’s scores between HTa and Baumann groups. Moreover, the HTa group was associated with shorter operation time and less radiation exposure in this investigation. Thus the HTa angle may be a convenient and reliable parameter, as the Baumann angle did, which could guide the reduction of supracondylar humerus fractures, especially when children are young.

## Data Availability

The data analyzed in this study are available from the corresponding author on reasonable request. All data in this study are included in this published article. The manuscript, including related data, figures, and tables has not been previously published and is not under consideration elsewhere.
